# Supplementary material for: Splice-Junction-Based Mapping of Alternative Isoforms in the Human Proteome
Source: Cell Rep. Author manuscript; Available in PMC 2020 Jan 15. (PMC6961840; doi:10.1016/j.celrep.2019.11.026)
Supplement: 2 [file NIHMS1546469-supplement-2.zip › DF1/PXD009737/TestisTrypsin/TestisTrypsin_75_TLK2_AGAAIASTSGASNNSSSMHVGNTGFIK.pdf]

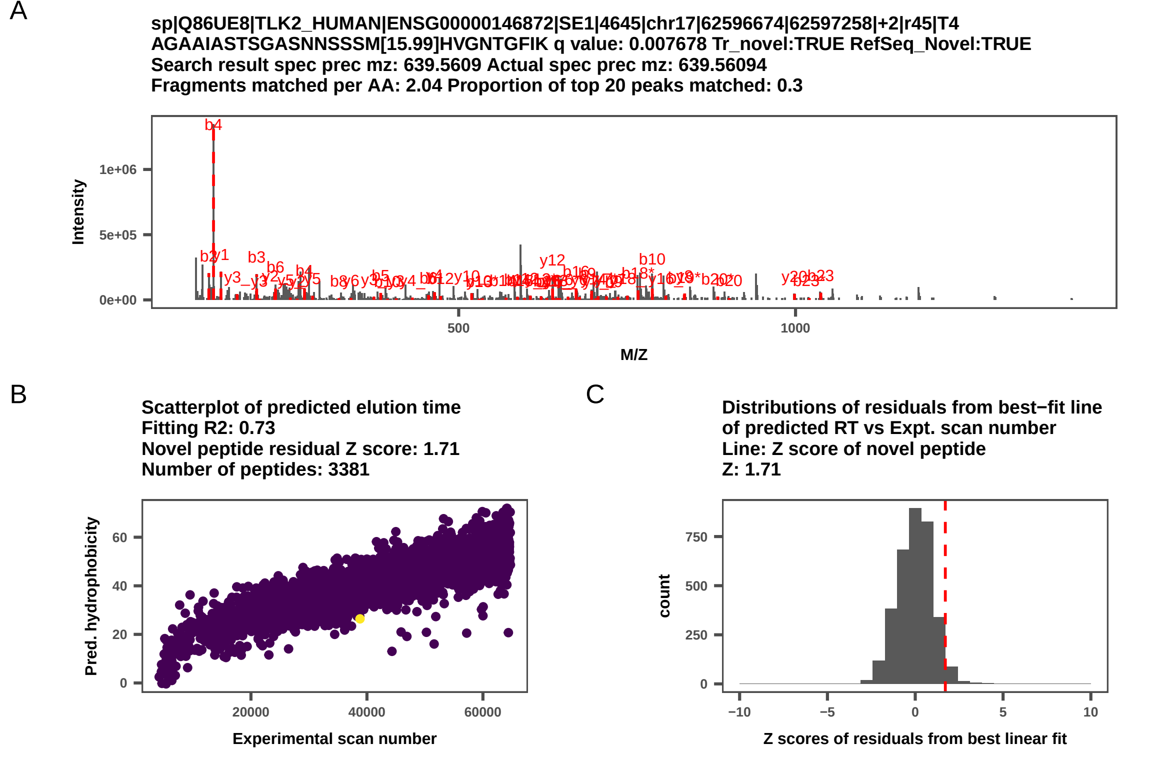
A
sp|Q86UE8|TLK2_HUMAN|ENSG00000146872|SE1|4645|chr17|62596674|62597258|+2|r45|T4
AGAAIASTSGASNNSSSM[15.99]HVGNTGFIK q value: 0.007678 Tr_novel:TRUE RefSeq_Novel:TRUE
Search result spec prec mz: 639.5609 Actual spec prec mz: 639.56094
Fragments matched per AA: 2.04 Proportion of top 20 peaks matched: 0.3
Intensity
1e+06
5e+05
0e+00
500
1000
M/Z
B
Scatterplot of predicted elution time
Fitting R2: 0.73
Novel peptide residual Z score: 1.71
Number of peptides: 3381
Pred. hydrophobicity
60
40
20
0
20000
40000
60000
Experimental scan number
C
Distributions of residuals from best−fit line
of predicted RT vs Expt. scan number
Line: Z score of novel peptide
Z: 1.71
count
750
500
250
0
−10
−5
0
5
10
Z scores of residuals from best linear fit
